# Protocolized Evaluation of Permissive Blood Pressure Targets Versus Usual Care (PRESSURE): Statistical Analysis Plan for the PRESSURE Trial

**DOI:** 10.1097/PCC.0000000000003961

**Published:** 2026-05-07

**Authors:** Tommaso Viola, Julia Broomhall, Zia Sadique, Paul Mouncey, David A. Harrison, David P. Inwald, for the Protocolized Evaluation of Permissive Blood Pressure Targets vs. Usual Care (PRESSURE) trial investigators on behalf of the Paediatric Critical Care Society Study Group (PCCS-SG), David Harrison

**Affiliations:** 1 Clinical Trials Unit, Intensive Care National Audit and Research Centre (ICNARC), London, United Kingdom.; 2 Department of Health Services Research and Policy, London School of Hygiene and Tropical Medicine, London, United Kingdom.; 3 Paediatric Intensive Care Unit, Addenbrooke’s Hospital, Cambridge University Hospitals NHS Foundation Trust, Cambridge, United Kingdom.

**Keywords:** health economics, mean arterial pressure, pediatric intensive care, randomized clinical trial, statistical analysis plan, vasopressor

## Abstract

**OBJECTIVE:**

To describe the prespecified analysis plan for Protocolized Evaluation of Permissive Blood Pressure Targets vs. Usual Care (PRESSURE), a trial comparing a permissive mean arterial pressure (MAP) target above the age-specific fifth centile with usual care in critically ill children.

**DESIGN:**

Pragmatic, open, multicenter, parallel group randomized controlled trial with integrated economic evaluation.

**SETTING:**

Twenty-one PICUs in the United Kingdom.

**PATIENTS:**

Infants and children older than 38 weeks corrected gestational age to 16 years, accepted to a participating PICU, on invasive mechanical ventilation (IMV) and receiving vasoactive drugs for hypotension.

**INTERVENTION:**

Adjustment of hemodynamic support to achieve a permissive MAP target greater than the fifth centile for age during IMV.

**MEASUREMENTS AND MAIN RESULTS:**

The primary outcome is a composite 30-day mortality and duration of IMV, and it will be analyzed with a two-sample rank-sum test. Components will also be analyzed separately. Sensitivity analyses will adjust for adherence, and subgroup analyses will test interactions with baseline covariates. Secondary analyses will compare mortality at various time-points, duration of survival, time to liberation from IMV, functional status changes, receipt of renal replacement therapy and length of stay. The health economic analysis will follow the intention-to-treat principle and report the mean (95% CI) incremental costs, quality-adjusted life years and cost-effectiveness up to 12 months.

**CONCLUSIONS:**

Results will be reported following this plan through peer-reviewed publications and conference presentations.

**RESEARCH IN CONTEXT**

- The previously published protocol article provides details on Protocolized Evaluation of Permissive Blood Pressure Targets vs. Usual Care (PRESSURE) trial rationale and delivery.
- This statistical analysis paper (SAP) describes the specific analysis strategies for testing the trial intervention.
- The SAP defines statistical and reporting procedures to ensure robust and unbiased evaluation of the trial objectives.

Hypotension (low blood pressure) is common among critically ill children. Interventions to treat hypotension include vasoactive drugs, IV fluids and central venous catheters. Although interventions are potentially lifesaving, there are also potential harms (1).

The Protocolized Evaluation of Permissive Blood Pressure Targets vs. Usual Care (PRESSURE) trial is a randomized controlled trial to evaluate the clinical and cost-effectiveness of permissive mean arterial pressure (MAP) target of greater than fifth centile for age compared with usual care. In this context, usual care is taken to mean blood pressure management without a protocolized MAP target, reflecting real-world practice in U.K. PICU.

PRESSURE is testing the hypothesis that the benefits of reduced exposure to hypotension treatments outweigh the risks of lower blood pressure. Further details of trial rationale and delivery can be found in the PRESSURE protocol article (2).

This article describes the proposed statistical and health economics analyses plan for the PRESSURE trial in accordance with published guidelines (3).

## METHODS

### Trial Design and Randomization

PRESSURE is a pragmatic, multicenter, parallel group, superiority randomized controlled trial. Eligible patients are randomized to either usual care or the permissive MAP target arm (age-dependent target). Allocation is by randomized permuted blocks with variable block lengths, stratified by site and age category (younger than 12 mo; 1 yr to younger than 3 yr; 3 yr or older).

### Sample Size

The sample size calculation used observational data from the Permissive vs. Restrictive Temperature Thresholds in Critically Ill Children with Fever and Infection (FEVER) study (4) which included 22 of 28 PICU wider National Health Service (NHS) units (*n* = 4126) and 6 months of eligible admissions.

Power estimates used 10,000 simulated trial datasets with distributions of mortality (13%) and duration of invasive mechanical ventilation (IMV) (log-normal distribution with mean = 7.8, sd = 9.5 d). To achieve 90% power, 1900 participants are required to detect a clinically meaningful mortality reduction of 2% (13–11%), and an 18-hour reduction in mean ventilator duration (7.8–7.1 d) in survivors, allowing for 10% withdrawal/refusal of deferred consent.

### Statistical Interim Analyses and Stopping Guidance

The internal pilot phase will be evaluated 6 months after the trial opens to recruitment against prespecified criteria (site opening, recruitment rate, and separation between groups in mean MAP and duration of vasoactives). A single-interim analysis will be carried out after recruitment 30-day follow-up of 950 patients. The composite primary endpoint will be analyzed in the intention-to-treat (ITT) population using a two-sample Wilcoxon rank-sum (Mann-Whitney) test, with early termination recommended if any one arm is shown to be superior with *p* value of less than 0.001 (Peto-Haybittle stopping rule).

### Timing of Final Analysis

The main analysis of the trial will be conducted after all patients randomized to the trial have completed 90-day follow-up, with one further update after 12-month follow-up.

### Research Ethics

The PRESSURE trial and consent procedures were approved by the East of United Kingdom–Cambridge South Research Ethics Committee (21/EE/0084; IRAS 289545) on May 10, 2021. The trial is registered with an International Standard Randomized Controlled Trial Number (20609635), and all procedures comply with the approved protocol and the Helsinki Declaration (1975, and later amendments).

## STATISTICAL PRINCIPLES

### CIs and *p* Values

The primary clinical endpoint will be tested for superiority. Secondary outcomes will be tested where specified or summarized descriptively. All tests will be two-sided with significance at *p* value of less than 0.05, and effect estimates presented with 95% CIs. No adjustments will be made for multiple testing.

### Adherence and Protocol Deviations

Exposure will be assessed and reported for each treatment group using: highest and mean MAP (recorded hourly until discontinuation of vasoactive agents for ≥ 24 hr or at PICU discharge); vasoactive receipt and duration to the end of first episode (defined as no vasoactive agents received for≥ 24 hr); dose-rate, total dose, total days on vasoactive agent at PICU discharge, number of treatment episodes; fluid balance; and urine output.

Vasoactive agent receipt will be summarized as number (%) of patients receiving each drug; other parameters will be reported as mean (sd) or median (interquartile range [IQR]), as appropriate. Norepinephrine equivalence will be calculated as previously described (5) (**Table 1**). Data on vasoactive drugs not included in the norepinephrine equivalence formula (e.g., milrinone) will be presented separately. Vasoactive agent infusions are recorded hourly and assumed to last one hour for total dose calculations.

**TABLE 1.** Conversion Method for Calculating Norepinephrine Equivalents (5)

Vasopressor	Unit	Conversion Factor for Norepinephrine Equivalent
Norepinephrine	μg kg^−1^ min^−1^	× 1
Epinephrine	μg kg^−1^ min^−1^	× 1
Dopamine	μg kg^−1^ min^−1^	/150
Phenylephrine	μg kg^−1^ min^−1^	× 0.1
Vasopressin	U min^−1^	× 2.5

### Protocol Adherence

The number (%) of patients deemed ineligible after randomization will be reported by treatment group, along with reasons for ineligibility.

Failure to discontinue vasoactive agents or reduce dose-rate once MAP is above the upper limit of the age-specific MAP target range for at least three consecutive hours in the permissive MAP target group defines a potential protocol deviation. Potential deviations will be adjudicated and may be deemed as an “acceptable” reason (**Table 2**), and the total number will be reported. Full adherence is defined as no “unacceptable deviations.” For each patient in the permissive MAP target group, the following adherence measures will be calculated: total time on vasoactive agents with MAP 1) within range, 2) above range, 3) greater than 5 mm Hg above the upper limit, and 4) below range, and summarized descriptively.

**TABLE 2.** Deviation Categories Following Adjudication Procedure

Unacceptable reasons
Deviation—clinician decision
Deviation—staffing issues/awareness
Unknown/not documented
Acceptable reasons
Patient developed an exclusion criterion postrandomization
Attempt to deliver protocol—volatile mean arterial pressure
Attempt to deliver protocol—monitoring issue
Patient met exclusion criteria at time of randomization
On vasoactives for an indication other than hypotension
Patient met an exclusion criterion at the time of randomization
Protocol not followed while outside of ICU

Categories may change before the final analysis.

### Analysis Population

Following the ITT principle, all randomized patients will be included in the primary analysis, excluding patients that have requested removal of all data. Analysis of secondary outcomes will additionally exclude those where consent to access medical records was withheld or unobtainable before the outcome timepoint.

## TRIAL POPULATION

### Recruitment

A Consolidated Standards of Reporting Trials flow diagram (6) will be completed to describe patients randomized and availability of data within each arm.

### Consent

The parent or legal guardian will be asked to provide consent as soon as appropriate after randomization. This model of consent, research without prior consent or “deferred consent” is increasingly used emergency care trials in many jurisdictions (7). Once given, consent can be withdrawn at any time.

If access to medical records is declined, data collection and any linkage to national datasets will not occur. Unless removal is explicitly requested, data collected up to refusal or withdrawal will be retained, and a minimal dataset including primary outcome data will be collected. If trial continuation is refused but access to medical records is granted, data collection and linkage will continue, and the patient may be included in the analysis as appropriate.

### Baseline Patient Characteristics

Baseline data are collected at randomization directly via the Case Report Form and at PICU admission via data linkage to the U.K. Paediatric Intensive Care Audit Network (PICANet). Baseline data will be summarized by group but not subjected to statistical testing.

## ANALYSIS

### Outcome Definitions

#### Primary Clinical Outcome

The primary outcome is a composite of death and duration of IMV at 30 days postrandomization. Patients who die on or before day 30 are assigned the worst score of 31. Surviving patients are scored by the total calendar days on IMV from day 1 (randomization) to day 30, including any IMV during readmissions to PICU.

#### Secondary Outcomes

Secondary outcome measures will include mortality at various time points, duration of survival from randomization to 12 months, time to liberation of IMV, change in functional status between PICU admission and PICU discharge, days of renal replacement therapy (RRT) to 30 days, length of PICU and hospital stay and health-related quality of life (HrQoL) at 12 months (**Table 3** for details).

**TABLE 3.** Secondary Outcomes

Mortality at PICU discharge	Death due to any cause before discharge from the PICU.
30-d/90-d/12-mo mortality	Death due to any cause before the relevant time point. ± 5 and ± 15 d of allowance will be considered if mortality at the exact time point is unknown, for the 90-d and 12-mo time point, respectively.
Duration of survival to 12 mo	Number of days where a patient is alive between randomization and 12 mo.
Time to liberation from invasive ventilation	Time from randomization to the start of the first continuous 48-hr period without IMV; deaths on IMV will be censored.
Functional status change between PICU admission and PICU discharge	Difference between pediatric Cerebral Performance Category and pediatric Overall Performance Category scores at admission and first PICU discharge for surviving patients.
Receipt of RRT at 30 d	Number of calendar days where RRT is received in PICU to day 30, among survivors to day 30.
Length of PICU stay	Hours until discharge plus any PICU readmissions by day 30.
Length of hospital stay	Days until discharge from any acute hospital.
Health-related quality of life at 12 mo	Assessed using child self- or parent-proxy completed age-appropriate Pediatric Quality of Life Inventory 4.0 (8), and the Child Health Utility 9D Index (9).

IMV = invasive mechanical ventilation, RRT = renal replacement therapy.

#### Cost and Health Economic Outcomes

These will include total costs at 12 months and Quality-adjusted Life Years (QALYs) at 12 months. Total costs will be calculated by valuing the resource use for delivering the interventions, length of stay in PICU and hospital, and wider NHS resource use up to 12 months.

QALYs at 12 months will be calculated from HrQoL and survival data. Incremental net monetary benefit (INB) at 12 months associated with permissive MAP target vs. usual care will be derived from incremental costs and QALYs at 12 months. INB will be calculated by valuing QALY gains at the National Institute for Health and Care Excellence (NICE) recommended (£20,000) willingness to pay for a QALY gain and subtracting incremental costs.

### Clinical Effectiveness Analysis Methods

#### Primary Outcome

The primary clinical outcome will be summarized by arm (median, IQR, graphically) and analyzed using a two-sample rank-sum (Wilcoxon-Mann-Whitney) test (10). The primary effect estimate will be the probabilistic index, the probability that intervention is superior to control for the composite outcome, and 95% CI. The separate components will also be presented by arm with effect sizes and 95% CIs, following established guidelines (11). Mortality will be analyzed using logistic regression, adjusting for age (younger than1 yr; 1 yr to younger than 3 yr; 3 yr or older), and site as a random effect. Duration of IMV will be analyzed using a two-sample rank-sum test.

Ordered logistic regression will estimate the proportional odds ratio, adjusting for baseline variables. The unadjusted OR will be presented for comparison. If data completeness allows, the following variables will be used in adjusted analyses: predicted log odds of mortality at randomization (Paediatric Index of Mortality, version 3 [PIM-3] score (12) [continuous; linear]); primary reason for admission (suspected/proven infection vs. other); comorbidities (neurologic vs. non-neurologic vs. none); age (continuous; restricted cubic splines with four knots at the 5th, 35th, 65th, and 95th percentiles); and site (random effect).

Baseline covariates were prespecified based on anticipated associations with the outcomes. Results will be reported as adjusted odds ratios with 95% CI.

#### Secondary Outcomes

Mortality outcomes will be compared using Fisher exact test. If suitable, logistic regression will be used, adjusted for baseline variables.

Duration of survival will be plotted as Kaplan-Meier survival curves by arm. An adjusted hazard ratio will be calculated using Cox proportional hazards regression.

Time to liberation from IMV will be reported as median time (with 95% CI) by arm using Kaplan-Meier methods. An adjusted hazard ratio will be calculated using Cox proportional hazards regression.

Change in functional status will be analyzed using adjusted linear regression, additionally adjusting for baseline status. Receipt of RRT at 30 days will be reported as median and IQR for patients surviving to day 30 and compared between groups using a Wilcoxon rank-sum test. Length of PICU and acute hospital stay will be compared using a Wilcoxon rank-sum test and reported by group using medians and IQRs in patients stratified by survival status. HrQoL score for survivors (12 mo) will be reported and a linear regression model adjusting for baseline variables will be applied to report incremental effect.

#### Subgroup Analyses

If sample sizes allow and following established guidelines (13, 14), subgroup analyses will test for interactions between treatment allocation and baseline covariates: age (continuous); infection (proven/suspected; none); comorbidities (neurologic vs. non-neurologic vs. none); PIM-3 score (continuous).

Where data completeness is below an acceptable threshold, multiple imputation may be used, or the relevant subgroup removed. The Instrument to Assess the Credibility of Effect Modification Analyses tool will assess the credibility of subgroup findings (15).

#### Sensitivity Analyses

The primary analysis will be repeated adjusting for adherence to allocated intervention (as a binary variable indicating any “unacceptable” deviation and/or as a continuous variable with percentage of treatment hours with no “unacceptable” deviation) using a structural mean model with an instrumental variable of allocated treatment to estimate the complier average causal effect of treatment (16). If convergence cannot be achieved, a per protocol analysis will be performed.

#### Handling of Missing Data

Where a single day of IMV data are missing but has been received on the previous and next day, it will be assumed that it was also received on the missing day. No IMV will be assumed on the day of hospital discharge unless otherwise recorded.

For the interim analysis, a complete case analysis will be presented (including only patients with complete data after the above assumptions). Additional sensitivity analyses will be performed under two different scenarios: IMV is provided on all missing days for patients in the permissive MAP target arm and not provided for all missing days for patients in the usual care arm, and vice versa.

For the final analysis where IMV data are missing, last observation carried forward will be applied while the patient is known to remain in PICU. For the adjusted analysis, levels of missingness of baseline variables will be assessed, and imputed if necessary.

Imputation will use the Multivariate Imputation using Chained Equations algorithm, with the model including all baseline variables included in the adjusted models, and the model including all outcome and baseline variables included in the sensitivity analysis. The number of imputations will be determined according to level of missingness. Models will be fitted in each imputed dataset and results combined using Rubin’s rules. If the levels of missingness in baseline data are deemed to be too high, the relevant baseline variable may be excluded from the analysis.

Analysis of change in functional status will use patients with non-missing data only and repeated with missing data imputed among patients known to be alive at those time points.

### Cost-Effectiveness Analysis Methods

#### Cost-Effectiveness Analysis

The cost-effectiveness analysis (CEA) will use patient-level data on resource use and outcomes from multiple sources: CRFs, PICANet data, and follow-up questionnaires (health services and HrQoL).

The CEA will adopt a health and personal health services perspective as recommended by NICE recommendations (17) and will measure use of resources required to deliver the intervention, length of stay in PICU and acute hospital, for the index admission and any readmissions within 12 months, and use of personal health services after acute hospital discharge within 12 months postrandomization. Resource use data will be valued using appropriate unit costs data from the NHS Payment by Results database, and unit costs of health and social care (Personal Social Services Research Unit), to calculate total costs up to 12 months.

QALYs will be calculated from responses to Pediatric Quality of Life Inventory (PedsQL) questionnaire which will be mapped onto the Child Health Utility 9D (CHU-9D) index score using an appropriate mapping function (18). The mapped CHU-9D index score will be combined with survival data to calculate QALYs. QALYs at 12 months postrandomization will be calculated by valuing each patient’s survival time by their HrQoL (as indicated by the mapped CHU-9D utility score) at 12 months, using the “area under the curve” approach (19). For 12 months survivors, QALYs will be calculated using the CHU-9D scores at 12 months, assuming HrQoL of zero at randomization, and a linear interpolation between randomization and 12 months. For nonsurvivors to 12 months, a zero QALY will be assumed for the 12-month period.

The CEA will follow ITT principle and will use appropriate regression methods. The interclass correlation coefficient (ICC) will be calculated which measures the proportion of the overall variation at the cluster level. If ICC greater than 10%, multilevel models (MLMs) will be used to handle clustering and avoid potential biases and incorrect inferences. The CEA model will adjust for key baseline covariates as per the primary clinical analysis. Missing data in health economic endpoints will be handled with appropriate multiple imputation methods, assuming the data are missing at random conditional on the observed data (20). On the imputed datasets the CEA will use a Bivariate Seemingly Unrelated Regression model to allow for correlation between costs and QALYs (21). The incremental results from multiply-imputed datasets will be summarized using Rubin’s rule (22).

The CEA will report the mean (95% CI) incremental costs, QALYs and net monetary benefit at 12 months. Results uncertainty will be summarized and represented on a cost-effectiveness plane and using acceptability curves. The CEA will report results for the overall patient group and subgroups as per the primary clinical analysis.

#### Sensitivity Analysis

Sensitivity analyses will be performed to check the robustness of assumptions made in the base case CEA in a number of ways. 1) HrQoL and QALY: An alternative CHU-9D mapping algorithm will be used to map the CHU-9D scores from the PedsQL responses. Alternative distributional assumptions for QALYs will be explored. 2) Cost data: Because of the likely skewed distribution of costs, several distributions will be explored. Implications of potential double-counting of costs across the different sources of resource use data will be assessed. 3) Complete case analysis. 4) Single vs. multilevel regression model: cost-effectiveness results across single vs. MLM that allows for clustering at sites will be checked.

### Safety

The numbers of serious adverse events (SAEs) and number and percentage of patients experiencing each SAE until critical care discharge will be reported by arm. The total number of patients experiencing one or more SAE will be compared between groups using Fisher exact test. Analysis of expected adverse events, as defined in the protocol article (2), will be also be descriptive and reported by arm.

### Statistical Software

Analyses will be conducted in Stata/MP 64-bit v18 or higher (StataCorp LLC, College Station, TX). Some secondary/sensitivity analyses may be carried out in R (R Foundation for Statistical Computing, Vienna, Austria).

## CONCLUSIONS

This statistical analysis paper prespecifies the analytic approaches to be adopted by the PRESSURE trial, before the recruitment target is reached. It defines outcomes and specifies statistical models and ensures transparency and reproducibility, providing a clear framework for interpreting the results of the trial.

## ACKNOWLEDGMENTS

The Protocolized Evaluation of Permissive Blood Pressure Targets vs. Usual Care (PRESSURE) trial investigators are: David Harrison, David Inwald, Joseph C. Manning, Paul Mouncey, Mark J. Peters, Padmanabhan Ramnarayan, Samiran Ray, Kathryn M. Rowan, Zia Sadique, Barnaby R. Scholefield, Dermot Shortt, Karen Thomas, and Helen Winmill. We thank our co-investigators and all members of the trial team, both at the Intensive Care National Audit and Research Council (ICNARC) and at the participating sites, for their invaluable contribution and support to the design, setup, and delivery of the PRESSURE trial.
